# *Streptococcus pyogenes* strains in Sao Paulo, Brazil: molecular characterization as a basis for StreptInCor coverage capacity analysis

**DOI:** 10.1186/s12879-015-1052-3

**Published:** 2015-08-05

**Authors:** Samar Freschi de Barros, Karine Marafigo De Amicis, Raquel Alencar, Pierre Robert Smeesters, Ariel Trunkel, Edilberto Postól, João Nóbrega Almeida Junior, Flavia Rossi, Antonio Carlos Campos Pignatari, Jorge Kalil, Luiza Guilherme

**Affiliations:** Heart Institute (InCor), School of Medicine, University of Sao Paulo, Sao Paulo, 01246-000 Brazil; Institute for Immunology Investigation, National Institute of Science and Technology, Sao Paulo, 01246-000 Brazil; Microbiology Laboratory of Clinical Hospital, School of Medicine, University of Sao Paulo, Sao Paulo, 01246-000 Brazil; Clinical Immunology and Allergy Division, School of Medicine, University of Sao Paulo, Sao Paulo, 01246-000 Brazil; Laboratoire de Génétique et Physiologie Bactérienne, Institute de Biologie et de Médecine Moléculaires, Faculté des Sciences, Université Libre de Bruxelles, Bruxelles, 1050 Belgium; Murdoch Childrens Research Institute, Parkville, 3052 Australia; Special Clinical Microbiolgy Laboratory (LEMC), Federal Univeristy of São Paulo/UNIFESP, São Paulo, Brazil; Laboratory of Immunology, Clinical Hospital, Heart Institute (HC-FMUSP), Av. Dr. Enéas de Carvalho Aguiar, 44, Sao Paulo, 05403-000 Brazil

## Abstract

**Background:**

Several human diseases are caused by *Streptococcus pyogenes*, ranging from common infections to autoimmunity. Characterization of the most prevalent strains worldwide is a useful tool for evaluating the coverage capacity of vaccines under development. In this study, a collection of *S. pyogenes* strains from Sao Paulo, Brazil, was analyzed to describe the diversity of strains and assess the vaccine coverage capacity of StreptInCor.

**Methods:**

Molecular epidemiology of *S. pyogenes* strains was performed by *emm*-genotyping the 229 isolates from different clinical sites, and PCR was used for superantigen profile analysis. The *emm*-pattern and tissue tropism for these M types were also predicted and compared based on the *emm-*cluster classification.

**Results:**

The strains were fit into 12 different *emm-clusters,* revealing a diverse phylogenetic origin and, consequently, different mechanisms of infection and escape of the host immune system. Forty-eight *emm*-types were distinguished in 229 samples, and the 10 most frequently observed types accounted for 69 % of all isolates, indicating a diverse profile of circulating strains comparable to other countries under development. A similar proportion of E and A-C *emm*-patterns were observed, whereas pattern D was less frequent, indicating that the strains of this collection primarily had a tissue tropism for the throat. *In silico* analysis of the coverage capacity of StreptInCor, an M protein-conserved regionally based vaccine candidate developed by our group, had a range of 94.5 % to 59.7 %, with a mean of 71.0 % identity between the vaccine antigen and the predicted amino acid sequence of the *emm*-types included here.

**Conclusions:**

This is the first report of *S. pyogenes* strain characterization in Sao Paulo, one of the largest cities in the world; thus, the strain panel described here is a representative sample for vaccine coverage capacity analysis. Our results enabled evaluation of StreptInCor candidate vaccine coverage capacity against diverse M-types, indicating that the vaccine candidate likely would induce protection against the diverse strains worldwide.

## Background

*Streptococcus pyogenes*, or Group A Streptococcus (GAS), is an exclusively human pathogen responsible for a broad variety of clinical manifestations ranging from pharyngitis and impetigo to invasive diseases, such as necrotizing fasciitis and toxic shock syndrome. Some strains can also trigger autoimmune diseases, such as acute rheumatic fever, rheumatic heart disease and glomerulonephritis [1]. GAS infections are the major cause of morbidity and mortality worldwide. The prevalence of severe GAS diseases is at least 18.1 million cases, which cause approximately 517,000 deaths per year [2].

M protein is a surface component of GAS and one of the main virulence factors due to its anti-phagocytic properties [3]. This protein contains a hyper variable amino terminal end that serves as substrate for gold standard *emm*-typing for strain identification. More than 220 different *emm*-types have been described [4]. Systematic epidemiological reviews clearly highlight significant differences in *emm*-type distribution across different regions of the world. Relatively limited numbers of *emm*-type are recovered from high-income settings, while a much higher diversity of strains circulates in low-income settings [5, 6]. A complementary typing system, *emm-*pattern typing, is based on the presence and arrangement of *emm* and *emm*-like genes located in the *mga* locus within the *S.pyogenes* genome. This classification is correlated with tissue tropism as follows: A-C *emm-*pattern isolates are usually recovered from the throat infections, D *emm-*pattern strains are usually isolated from the skin (impetigo), and E *emm*-patterns are recovered from both biological sites [7, 8].

Sanderson-Smith et al. recently proposed a functional classification of the *emm*-types in clusters according to the phylogenetic origin and microbiological characteristics of the strain. The cluster classification enabled comparison between strains and serves as a tool for vaccine development [9].

GAS contains numerous genes encoding virulence factors, such as streptococcal pyrogenic exotoxins (Spe proteins). These proteins constitute a family of bacterial toxins with powerful mitogenic effects on T cells expressing a particular Vβ domain of the T cell receptor molecule, inducing non-specific polyclonal activation of the immune system by binding directly to class II MHC molecules [10]. Several studies have reported that Spe exotoxin content is correlated with *emm*-types and associated with clinical manifestations [11–13]. Spe exotoxins most likely contribute to the severity of GAS infections. However, the exact molecular mechanism involved in specific pathologies is still not understood [14].

To date, no anti-streptococcal A vaccine is available; however, several candidates based on both N- and C- terminal portions of the M protein are in different stages of development [15]. Briefly, the 30-valent is based on the highly variable amino-terminal region of the M protein [16], and the J8 candidate vaccine a construction of minimal B-cell epitope from the C-repeat region [17].

StreptInCor candidate vaccine is based on amino acid sequences of the conserved region of the M5 protein. This candidate vaccine, in contrast to the others, contains both B and T cell epitopes to provide a strong protective immune response [18].

Although GAS infections are common in several regions of Brazil, only a few studies on the prevalence, *emm*-type profiles and virulence factors of the strains are available [19–21]. Here, we described the *emm*-type and superantigen profile of the most prevalent strains in Sao Paulo and assessed the theoretical coverage vaccine.

## Methods

### *S.pyogenes* strain collection

GAS isolates were obtained from patients treated at the Clinical Hospital, School of Medicine, University of Sao Paulo, Sao Paulo, and the Special Clinical Microbiology Laboratory (LEMC), Federal University of Sao Paulo, Sao Paulo, Brazil, between 2001 and 2008. The bacterial samples were defined according to their isolation sites (skin, throat and other invasive sites).

Institutional Review Board (IRB) approval was obtained from the Heart Institute Ethics Committee (CAPPesq; approval number-0646/07) at the University of Sao Paulo. Patient informed consent was waived because this study is a retrospective analysis of strains from a microbiology collection.

The GAS diagnostic criteria were based on beta hemolysis in blood agar and sensitivity to bacitracin. Then, the specimens were cultured on sheep blood agar (Vetec, Brazil), followed by growth in Todd-Hewitt broth (Himedia, India) until OD_600_ of 0.4 and stored at −80 **°**C.

### DNA isolation, *emm*-typing, patterning and *emm*-cluster distribution

The genomic DNA extraction, *emm-*gene PCR amplification and sequencing and *emm*-type identification were performed according to the protocol described by the CDC (http://www.cdc.gov/ncidod/biotech/strep/strepblast.html) using the primers MF2 and MR1 for amplification and sequencing, respectively, as previously described [19]. The *emm*-pattern for each *emm*-type was deduced using the table of correspondence provided by a recent multi-center study [4]. The *emm*-cluster classification of the strains identified in this study was based on the new functional classification recently proposed by Sanderson-Smith et al. [9].

### Superantigen profile

To identify the superantigens each gene carried by strain, PCR reactions were performed using specific primers and singleplex PCR as previously described for *speA*, *speC, speG*, *speH, speI*, *speJ*, *ssa* [13] and *smeZ* [12]. *speB* (cysteine protease) was used as a positive control in our PCR reaction.

### Statistical analysis

The Simpson Reciprocal Index (1/D) of 1 corresponds to a theoretical situation in which only one *emm*-type/cluster is recovered, representing the lowest diversity possible. The maximum Simpson Reciprocal Index corresponds to the total number of *emm*-type/cluster recovered in one area. Higher values indicate greater diversity. A Simpson Index was calculated using the following formula: D = ∑ (n/N) 2, where “n” is the total number of isolates of a given *emm*-type or belonging to a given cluster and “N” is the total number of isolates of all the *emm*-types/clusters recovered in an area [22, 23]. Confidence intervals were calculated as previously described [24].

### M protein sequence analyses

M proteins complete sequences and C repeat annotation from each *emm*-type included in this study were derived from previous study [4]. Multiple proteic alignments were obtained using Muscle software as implemented in Geneious® version R8.

## Results

### *emm-*types

**Fig. 1:**
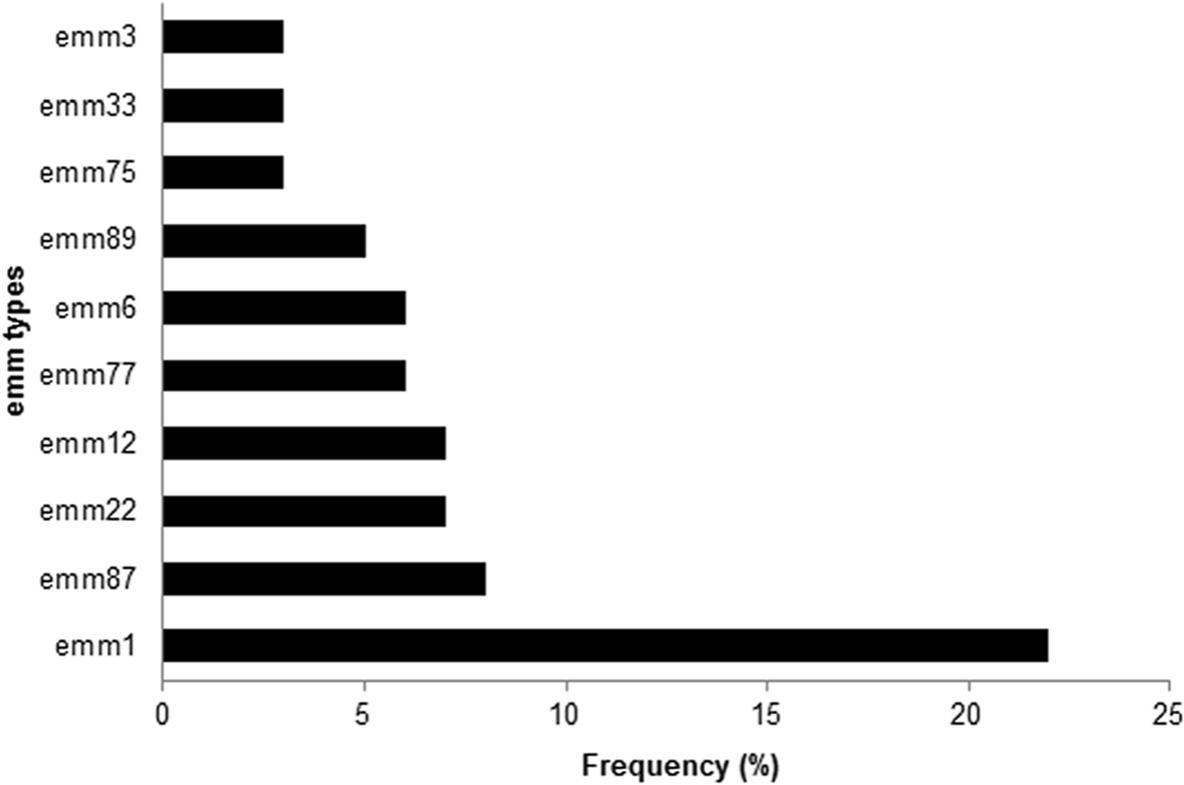
Frequency of *emm-*types. A total of 48 *emm*-types were represented in the collection. Abbreviation: GAS, group A streptococcus

The distribution of *emm*-types among the 229 GAS isolates is described in Table 1. The clinical origin was known for 214 isolates. Most samples were associated with invasive infection (n = 123, 57 %), whereas the remaining samples were recovered from throat (n = 57, 27 %) and skin infections (n = 34, 16% ). Forty-eight different *emm*-types were identified. The most frequent *emm*-types were *emm*1 (22 %), *emm*87 (8 %), *emm*22 (7 %), *emm*12 (7 %), *emm*77 (6 % ), *emm*6 (6 % ), *emm*89 (5 %), *emm*33 (3 %), *emm*75 (3 %) and *emm*3 (3 %) (Fig. 1). Taken together, these *emm*-types accounted for 69 % of the GAS isolates. To better understand the strain diversity present in our study, and its likely consequence for multivalent vaccine coverage, we have calculated the reciprocal Simpson index of diversity which results was 12.7 (95 % CI, 10.1-17.0).

**Table 1:** Distribution of *emm*-types among 229 GAS isolates obtained during the 2004-2008

*emm* types	N/% of each strain per year									N/% among total
	2001	2002	2003	2004	2005	2006	2007	2008	Unknown	
1	3/18.8	5/22.7	11/25.6	6/40	6/15.0	1/3.4	6/16.7	4/40.0	8/44.4	50/21.8
87	1/6.3		5/11.6	1/6.7	1/2.5	3/10.3	5/13.9	1/10.0	1/5.6	18/7.8
22	5/31.3	2/9.0	2/4.7	1/6.7	1/2.5	1/3.4		3/30.0	2/11.1	17/7.4
12			2/4.7	2/13.4	6/15	1/3.4	4/11.1			15/6.5
77		2/9.0	4/9.3		3/7.5	1/3.4	2/5.6	1/10.0	1/5.6	14/6.1
6			1/2.3		4/10.0	6/20.7	2/5.6			13/5.7
89			1/2.3			3/10.3	3/8.3	1/10.0	3/16.7	11/4.8
75	2/12.5	1/4.5	1/2.3	3						7/3.0
33			0		3/7.5	2/6.9	1/2.8		1/5.6	7/3.0
3		1/4.5	2/4.7			3/10.3				6/2.6
183			0		4/10.0	1/3.4				5/2.1
78		1/4.5	4/9.3							5/2.1
53			0				3/8.3		1/5.6	4/1.7
64		1/4.5	0		1/2.5		1/2.8			3/1.3
92		1/4.5	1/2.3		1/2.5					3/1.3
108		2/9.0					1/2.8			3/1.3
95							3/8.3			3/1.3
41	1/6.3			1/6.7						2/0.9
4	1/6.3					1/3.4				2/0.9
44					1/2.5		1/2.8			2/0.9
49			1/2.3		1/2.5					2/0.9
58		2/9.0	0							2/ 0.9
59		0	1/2.3		1/2.5					2/0.9
73		1/4.5	0		1/2.5					2/0.9
80			1/2.3			1/3.4				2/0.9
101			0		1/2.5				1/5.6	2/0.9
102			1/2.3			1/3.4				2/0.9
115						2/6.9				2/0.9
127					1/2.5		1/2.8			2/0.9
99		1/4.5			1/2.5					2/0.9
Other^a^	3/18.8	2/9.0	5/11.6	1/6.7	3	2/6.9	3/8			19/8.3
Total/year	16/100	22/100	43/100	15/100	40/100	29/100	36/100	10/100	18/100	229/100

^a^ *emm*-types with one sample only: 57, 63, 66, 67, 68, 71, 76, 83, 85, 86, 88, 90, 94, 119, 122, 184, 186, 193. N/percentage: number and percentage of each strain per year

### *emm*-pattern and *emm*-cluster distribution

We inferred the *emm*-pattern for 213 of 214 *emm*-types, except for *emm127* (previously named st223). Pattern E and A-C *emm*-types were present at similar proportions (43 and 38 %), whereas pattern D strains were less frequent (18 %).

The strains were classified according to the *emm*-clusters, and the strains fit into 12 of 19 different *emm-*clusters. Most strains belonged to *emm*-cluster A-C3 (21 %), followed by E4 (20 %), E3 (13 %), D4 (12 %), single protein cluster clade Y (9 %), A-C4 and E6 (7 %), A-C5 and E1 and E2 (3 %), D2 and D5 (1 %) (Table 2).

**Table 2:** *emm*-cluster classification

*emm* type	Number of samples	*emm* cluster	% *emm* cluster
1	50	A-C3	21
12, 193	16	A-C4	7
3	6	A-C5	3
71	1	D2	1
33, 41, 53, 64, 80, 83, 86, 101, 108, 119, 186	27	D4	12
184	1	D5	1
4, 78	7	E1	3
66, 68, 76, 90, 92	7	E2	3
44, 49, 58, 87, 183	30	E3	13
22, 73, 77, 88, 89, 102	47	E4	20
59, 63, 67, 75, 85, 94, 99, 115	17	E6	7
6, 57, 95, 122	18	single protein cluster clade Y	9

The *emm*-types obtained fit into 12 different *emm-*clusters: A-C3 (21 %), E4 (20 %), E3 (13 %), D4 (12 %), single protein cluster clade Y (9 %), A-C4 and E6 (7 %), A-C5 and E1 and E2 (3 %), D2 and D5 (1 %)

### Superantigen profile

The superantigen gene encoding profile was analyzed in 219/229 isolates (96 %). The chromosomally located superantigens genes *smeZ*, *speG*, and *speJ* were present in 219 (95.6 %), 201 (88 %) and 79 (35 %) isolates, respectively. The *speG* and *smeZ* genes were present at high frequencies in all strains, whereas *speJ* was absent or uncommon in diverse *emm*-types and presented a higher frequency only in *emm*1, *emm*33 and *emm*87 (n = 72, 86 %). Among the phage-encoded genes, *speC* was the most prevalent (n = 109, 48 %), followed by *ssa* (n = 61, 27 %), *speA* (n = 43, 19 %), *speH* (n = 37, 16 %), and *speI* (n = 31, 14 %). Among the most prevalent *emm*-types, *speA* was present in *emm*3 (100 %) and *emm*1 (62 %) but in only one sample of *emm*6. The *emm*-type *speC* was associated with all strains but was less frequent in *emm*1, *emm*3, *emm*183 and *emm*75 (n = 12, 33 %) and more frequent in the remaining strains (n = 38, 93 %). Additionally, *speI* was absent or less frequent in most samples, except for *emm*12 and *emm*183 (53 % and 60 %, respectively). Finally, *speH* was also absent or uncommon in most *emm*-types and occurred at a higher frequency only in *emm*183, *emm*12 and *emm*78 (n = 40, 72 %), and *ssa* was absent in only one isolate, with a frequency range of 7-86 % (Table 3).

**Table 3:** Superantigen profile of the most frequent *emm*-types identified in Sao Paulo, Brazil

*emm* type	Number of samples	Superantigens number (%)							
		*speG*	*smeZ*	*speC*	*ssa*	*speH*	*speI*	*speJ*	*speA*
*emm*1	48	45(94)	48(100)	6(12)	10(21)	1(2)	3(6)	39(81)	30(62)
*emm* 53	4	4(100)	3(75)	3(75)	1(25)	1(25)	1(25)	1(25)	-
*emm33*	7	7(100)	6(86)	4(57)	5(71)	2(29)	1(14)	6(86)	-
*emm* 22	16	14(87)	16(100)	13(81)	11(69)	2(12)	1(6)	-	-
*emm* 12	15	13(87)	15(100)	14(93)	1(7)	8(53)	8(53)	-	-
*emm*78	5	4(80)	5(100)	3(60)	1(20)	2(40)	-	1(20)	-
*emm* 6	12	12(100)	12(100)	83(77)	2(17)	-	1(8)	1(8)	1(8)
*emm* 87	18	13(72)	18(100)	15(83)	13(72)	-	-	13(72)	-
*emm* 77	13	6(46)	11(85)	5(38)	2(15)	-	-	1(8)	-
*emm* 89	11	11(100)	11(100)	8(82)	2(18)	-	-	4(36)	-
*emm3*	6	6(100)	5(83)	1(17)	3(50)	-	-	-	6(100)
*emm* 183	5	5(100)	5(100)	1(20)	-	4(80)	3(60)	-	-
*emm* 75	6	6(100)	6(100)	2(33)	3(50)	-	-	-	-

### Vaccine coverage

**Fig. 2:**
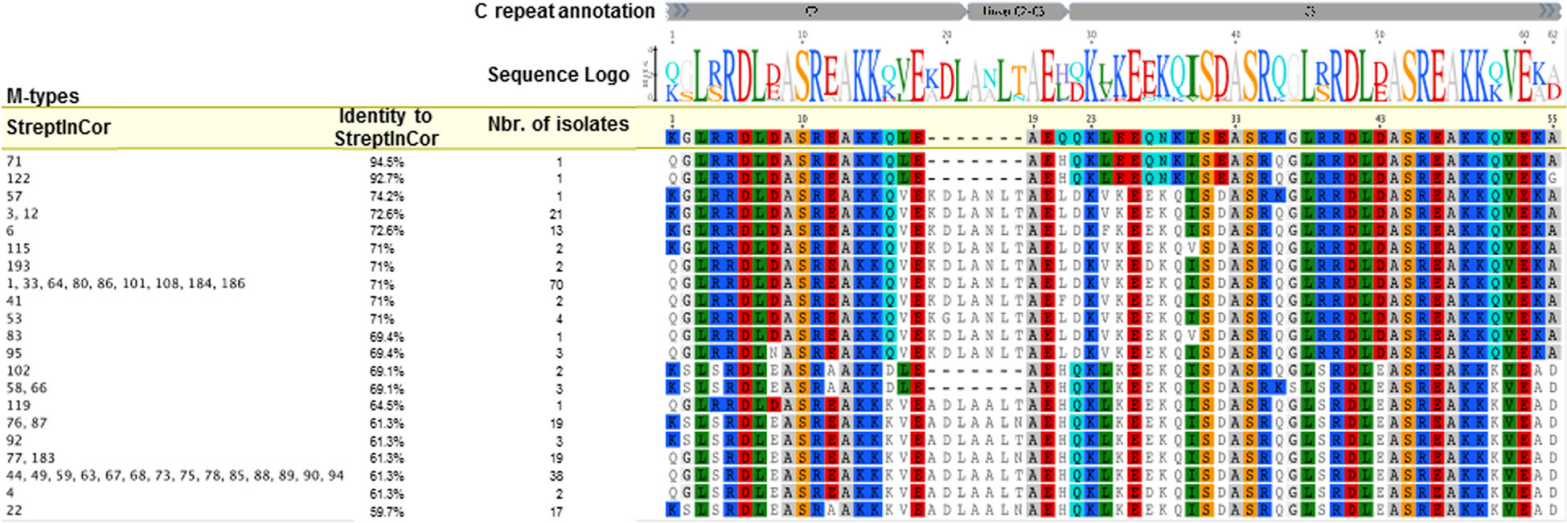
*In silico* analysis of StreptInCor coverage capacity. Amino acid sequence alignment of StreptInCor candidate vaccine with the 46 *emm*-types identified here (the complete M protein sequence was missing for both *emm127* and *emm99*)

Theoretical vaccine coverage capacity of StreptInCor candidate vaccine was accessed considering the amino acid sequence alignment with the M protein C-terminal region for the 46 *emm*-types identified here (the complete M protein sequence was missing for both *emm*127 and *emm*99). The identities ranged from 94.5 % to 59.7 % (mean of 71 %). Some *emm*-types presented with an insertion of 7 amino acid residues in their sequences, as previously described (Fig. 2).

## Discussion

*Streptococcus pyogenes* is an important human pathogen responsible for several invasive and non-invasive diseases in Brazil and worldwide. In this study, we characterized 229 invasive and non-invasive *Streptococcus pyogenes* samples from patients treated at the Clinical Hospital in Sao Paulo, Brazil. Great diversity of *emm*-types was observed. Forty-eight *emm*-types were observed in the 229 samples, with the 10 most frequent *emm*-types accounting for 69 % of all isolates. In terms of GAS strain diversity, a Simpson Reciprocal Index of 1 corresponding to a theoretical situation where only one *emm*-type/cluster has been recovered, representing the lowest diversity possible. The maximum value of the Simpson Reciprocal Index corresponds to the total number of *emm-*type/cluster recovered in one area. The higher the value is, the greater the diversity. The reciprocal Simpson index of diversity found in this study was relatively low (12.7) when compared to the index of 26.72 for Brasilia (in the central region of Brazil) [19]. On the other hand, our results were similar to those reported for high incomes suburbs from Salvador, in northeastern Brazil [20].

The distribution of the strains identified in this study is comparable to those found in other countries, particularly in high-income countries in Asia, the Middle East and Latin America, in which *emm*1 and *emm12* were the most common types, as reviewed by Steer [6]. Interestingly, *emm*1, *emm*12 and *emm*89 have also been found in various studies conducted recently in several countries in Europe and China; these types were frequently correlated with invasive and/or noninvasive isolates [25]. *emm*77 had a high frequency in the invasive isolates found here. In addition, this strain has been associated with non-invasive diseases in Germany [26] and was found in both invasive and non-invasive isolates in Spain [12]. Among the 229 isolates, E and A-C *emm*-patterns were found in similar proportions, whereas pattern D was less frequent. Interestingly, studies from Brasilia, in the Central region of Brazil [19], revealed a higher proportion of E and D patterns (51 % and 36 %, respectively), whereas A-C patterns was rarely observed (9.5 %). The data demonstrate the variability of streptococcal strains in Brazil, which may be related to socio-economic differences and can be extended to other countries in which there are also social disparities.

Other factors that play a role in the clinical manifestation of *S. pyogenes* infection may be due to the associations between *emm*-types and superantigens.

In this study, the chromosomally encoded genes *smeZ* and *speG* occurred at high frequency in nearly all isolates (95.6 and 88 %, respectively); both were present in all *emm*-types at high frequencies (<70 %), except *speG* in *emm*77(43 %), in according with a variety of others studies [12, 27–29].

The other chromosomal gene, *speJ*, was present in only 35 % of isolates and was absent in diverse *emm*-types, similar to others studies [12, 29, 30].

Among the phage-encoded genes, *speC* was the most prevalent, detected in 48 % of the isolates, followed by *ssa* (27 %), *speA* (19 %), *speH* (16 %), and *speI* (14 %). The *speC*, *ssa*, *speH* and *speI* genes presented similar frequencies to those found in others studies, whereas the *speA* gene generally had a lower frequency in our samples [25, 30, 31]. *speA* was present in *emm*3 (100 %), *emm*1 (62 %) and only one sample of *emm*6. The *speA* genes has been commonly detected among 1 isolate in several studies [32].

Currently, no anti-streptococcal vaccine is available in animal models of streptococcal disease, despite extensive efforts. Some models of anti-streptococcal vaccines are in different stages of development. Among them, the 30-valent contains short peptides from the highly variable amino-terminal region of the M protein [16], and the J8 vaccine candidate comprises a 12 amino acid minimal B-cell epitope from the C-repeat region flanked by 16 amino acids of a yeast DNA-binding protein conjugated to the diphtheria toxoid [17].

The vaccine candidate developed by our group, called StreptInCor, is based on the M5 protein C-terminal region [18], specifically the C2 and C3 region that is conserved among serotypes. Through *in silico* analysis with predicted amino acid sequence alignment, StreptInCor candidate vaccine had high sequence identity with 46 of the 48 *emm*-types described here (identity ranged from 94.5 % to 59.7 %, mean of 71 %), which is an important property for the probability of protection. In previous data, we described the structural, chemical, and biological properties of the StreptInCor peptide and demonstrated that the molecule is stable, which is an important property for a vaccine candidate. The possibility of the StrepInCor vaccine candidate epitope being processed by antigen-presenting cells (APCs) generating diverse peptides has also been previously demonstrated. The approach resulted in the observation that the vaccine epitope could be recognized by any individual, thus enabling a broad coverage capacity to trigger specific immunity [33].

The efficacy of this vaccine in animal models was evaluated in inbred and outbred mice, and a strong humoral response with high IgG production was observed [18]. Immunized Swiss mice challenged with the *emm*1 strain had a survival rate of 87 % at 21 days compared with lower survival in controls (53 %) [34].

Similar results have been observed in HLA class II transgenic mice, which also presented a specific and long-lasting immune response without developing deleterious reactions after one year. These results indicated that StreptInCor is a safe candidate vaccine [35].

In addition, the four most common *emm*-types included here (*emm*1, *emm*12, *emm*22 and *emm*87) were opsonized by StreptInCor-induced antibodies [36]. The strains identified here were fit into 12 of the 19 different *emm-*clusters and exhibited diverse phylogenetic origin and consequently different mechanisms of infection and resistance to escape the host immune system, supporting the hypothesis that StreptInCor vaccination would likely protect against infection caused by strains from different *emm*-clusters.

## Conclusions

This is the first study investigating the epidemiology of streptococcal strains in Sao Paulo, one of the largest cities in the world. These data enabled evaluation of the StreptInCor candidate vaccine coverage capacity against diverse M-types, indicating that the vaccine candidate would likely induce protection against the diverse strains observed worldwide.
